# Proopiomelanocortin (POMC) and psychodermatology

**DOI:** 10.1002/ski2.201

**Published:** 2023-04-07

**Authors:** George W. M. Millington, Hannah E. Palmer

**Affiliations:** ^1^ Dermatology Department Norfolk and Norwich University Hospital Norwich UK; ^2^ Norwich Medical School Norwich UK

## Abstract

Psychodermatology is the crossover discipline between Dermatology and Clinical Psychology and/or Psychiatry. It encompasses both Psychiatric diseases that present with cutaneous manifestations (such as delusional infestation) or more commonly, the psychiatric or psychological problems associated with skin disease, such as depression associated with psoriasis. These problems may be the result either of imbalance in or be the consequence of alteration in the homoeostatic endocrine mechanisms found in the systemic hypothalamic‐pituitary‐adrenal axis or in the local cutaneous corticotrophin‐releasing factor‐proopiomelanocortin‐corticosteroid axis. Alteration in either of these systems can lead to immune disruption and worsening of immune dermatoses and vice‐versa. These include diseases such as psoriasis, atopic eczema, acne, alopecia areata, vitiligo and melasma, all of which are known to be linked to stress. Similarly, stress and illnesses such as depression are linked with many immunodermatoses and may reflect alterations in the body's central and peripheral neuroendocrine stress pathways. It is important to consider issues pertaining to skin of colour, particularly with pigmentary disorders.

## Graphical abstract

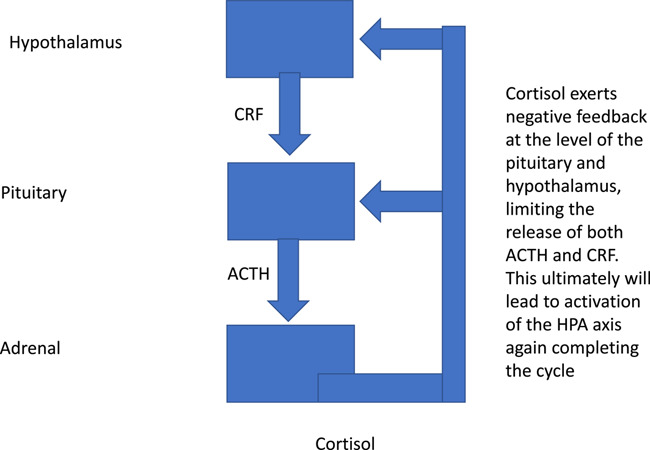

1

**What's already known about this topic?**

- The involvement of central and peripheral proopiomelanocortin pathways in acute and chronic stress is well‐established.

**What does this study add?**

- This opinion looks for evidence linking stress with predominantly inflammatory skin disease.

## 2 INTRODUCTION

### 2.1 HPA axis and POMC regulation

**FIGURE 1:**
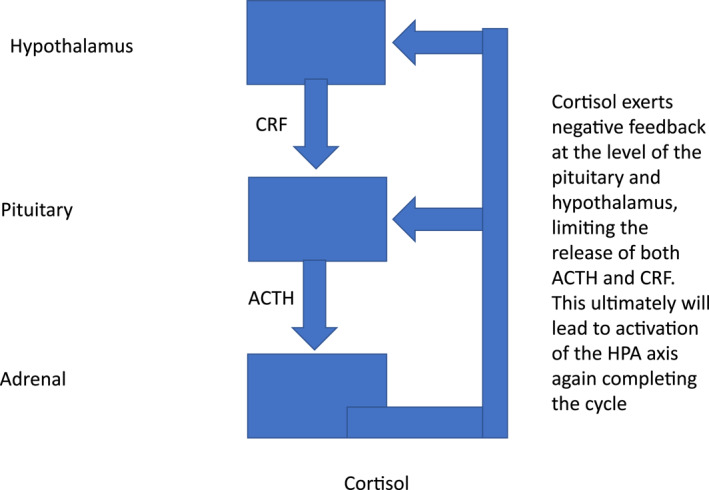
Stylised hypothalamo‐pituitary‐adrenal (HPA) axis.

**FIGURE 2:**
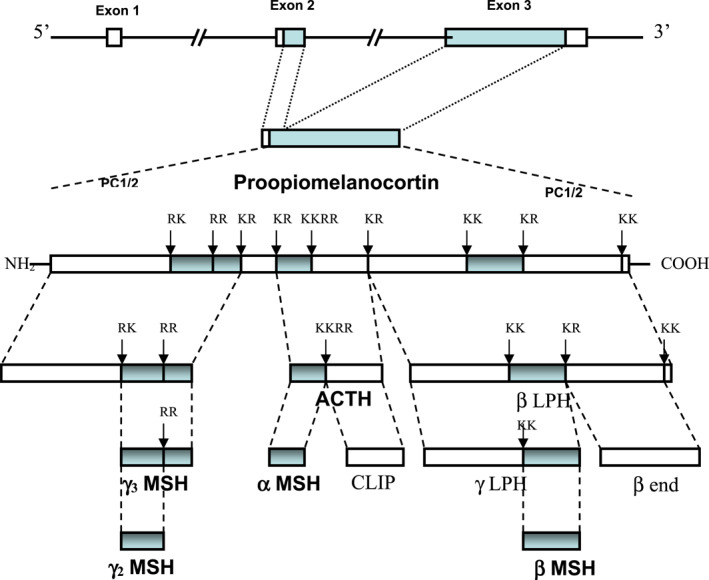
Gene structure and post‐translational processing of POMC. POMC in mammals consists of three exons, of which exons 2 and 3 are translated. PC1/2 break the parent POMC peptide into successively smaller peptides by cleavage at paired dibasic amino acid residues consisting lysine (K) and/or arginine (R). The final products are generated in a tissue specific manner, for example, α‐MSH and ACTH are not produced by the same cells in the pituitary. They also involve additional enzymatic post translational modifications, such as the acetylation of α‐MSH. The final products include the melanocortins (MSHs and ACTH), β‐end and CLIP. There are intermediate peptides whose biological function remains unclear, such as β‐LPH, γ‐LPH. CLIP, corticotrophin‐like intermediate peptide; PC1/2, prohormone convertases 1 and 2; POMC, proopiomelanocortin; α‐MSH, α‐melanocyte‐stimulating hormone; β‐end, β‐endorphin; β‐LPH, β lipotrophin; γ‐LPH, γ lipotrophin.

**FIGURE 3:**
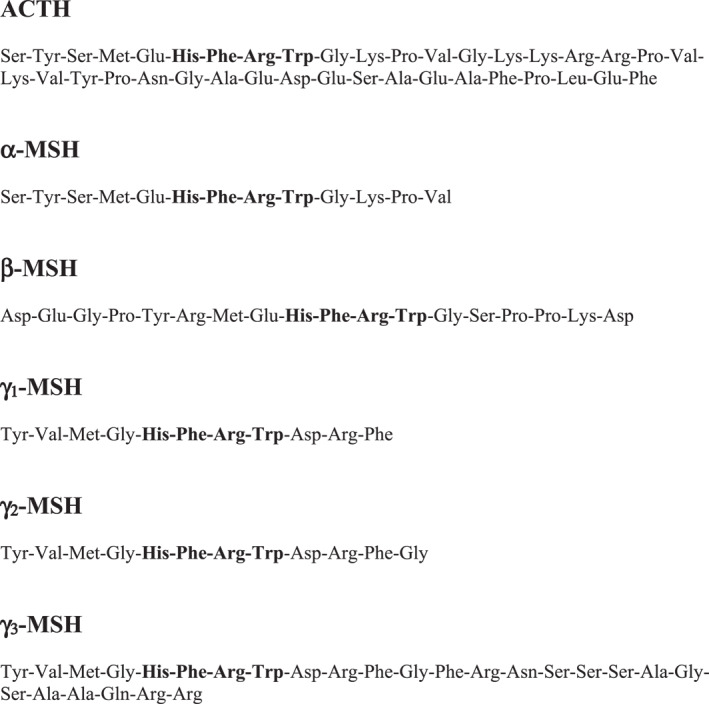
Natural melanocortins. These include the various MSH peptides and corticotrophin (ACTH). All share the common structure, His‐Phe‐Arg‐Trp (in bold) which facilitates binding to the MCR's. ACTH also contains the cleavage site, Lys‐Lys‐Arg‐Arg, which allows it to form α‐MSH and CLIP (see Figure 2). CLIP, corticotrophin‐like intermediate peptide; MCR's, melanocortin receptors; MSH, melanocyte‐stimulating hormone.

The hypothalamo–pituitary–adrenal (HPA) axis is a complex set of positive and negative feedback mechanisms between the hypothalamus, anterior pituitary and adrenal glands (Figure 1).^1^ Other organs, such as the skin, thymus gland and other immunocytes may also be involved.^1^, ^2^, ^3^, ^4^ These positive and negative feedback systems work in a neuroendocrine‐immunoregulatory manner to control several processes such as immunity, fertility and the body's resilience to both acute and chronic stress.^1^, ^2^, ^5^ The process by which the HPA axis continues to have a homoeostatic role depends on the release and uptake of several important peptide or steroid factors.^1^, ^2^, ^3^, ^4^ The hypothalamic part of the HPA axis consists principally of neurones that secrete corticotrophin‐releasing factor (CRF) and secondarily arginine vasopressin (AVP).^1^, ^2^, ^3^, ^4^, ^5^, ^6^, ^7^, ^8^ CRF/AVP will, in turn, act on the adenohypophysis (anterior pituitary gland) to elicit the synthesis and secretion of adrenocorticotrophin (ACTH) into the bloodstream.^1^, ^2^, ^3^, ^4^, ^5^, ^6^, ^7^, ^8^ ACTH is exclusively produced from proopiomelanocortin (POMC), which is predominantly synthetized in the corticotroph and melanotroph cells of the anterior and intermediate lobes of the pituitary gland, the arcuate nucleus of the hypothalamus and to a lesser extent the skin.^1^, ^2^, ^3^, ^4^, ^5^, ^6^, ^7^, ^8^, ^9^, ^10^ Circulating ACTH then induces the adrenal gland to produce and release corticosteroids (CSs), such as cortisol.^1^, ^2^, ^3^, ^4^, ^5^, ^6^, ^7^ These circulating CSs regulate the vast array of processes influenced by the HPA axis and also produce a negative feedback loop on the HPA axis via activation of the glucocorticoid receptor in the brain to shut down CS production.^1^, ^2^, ^3^, ^4^, ^5^, ^6^, ^7^ ACTH may also target the thymus to produce glucocorticoids independently of the adrenal, which then feedback at the level of the hypothalamus and pituitary.^6^ Another mechanism the thymus can influence the HPA axis is through alteration in the secretion of thymic peptides, which then contribute to the feedback loop centrally at the level of the arcuate nucleus and adenohypophysis.^7^ POMC, a precursor protein, yields many biologically active peptides via a series of enzymatic steps (PC1, PC2 and PC3) in a tissue‐specific manner (Figure 2).^1^, ^2^, ^3^, ^4^, ^5^, ^6^, ^7^, ^8^, ^9^, ^10^ In the anterior pituitary lobe, PC1/3 is responsible for the post‐translational cleavage that generates 16‐kDa N‐POMC, ACTH, and β‐lipotrophin.^1^, ^2^ In the intermediate pituitary lobe and arcuate nucleus of the hypothalamus, a more complex processing of POMC, including PC2 activity along with other enzymes, generates more active peptides, such as α‐melanocyte‐stimulating hormone (α‐MSH) and β‐endorphin (Figure 3).^1^, ^2^, ^3^ PC2 activity may be more important in the cutaneous processing of POMC.^10^

### 2.2 The MCRs

The MSHs and ACTH bind to the extracellular G‐protein coupled (GPCRs) melanocortin receptors (MCRs) of which there are five subtypes (Table 1).^1^, ^2^, ^3^, ^4^, ^5^, ^6^, ^7^, ^10^, ^11^ β‐endorphin binds to the opiate family of GPCRs separately.^9^ The MC3R and MC4R show widespread expression in the central nervous system (CNS), whilst there is low level expression of MC1R and MC5R.^3^, ^11^ In the CNS, cell bodies for POMC are mainly located in the arcuate nucleus of the hypothalamus and the nucleus tractus solitarius of the brainstem.^1^, ^2^, ^3^ Both areas have well defined functions relating to appetite and food intake.^3^, ^11^, ^12^ Two (MC1R and MC5R) show widespread cutaneous expression.^4^, ^10^ ACTH and α‐MSH bind to MC1R to influence both pigmentation and the immune system.^4^, ^10^ MC5R regulates the sebaceous glands.^4^, ^10^ The MC2R is the ACTH receptor, predominantly located in the adrenal gland and skin in humans.^1^, ^2^, ^4^, ^10^ In addition to MC2R, which is highly specific for ACTH, other MCRs (MCR1, MCR3, MCR4, and MCR5) can bind to ACTH and other POMC‐derived peptides.^1^, ^2^, ^3^, ^4^, ^5^, ^6^, ^7^, ^10^, ^11^, ^13^, ^14^, ^15^, ^16^, ^17^, ^18^ The five known MCRs have established biological functions.^13^ With the exception of MC2R, these receptors can behave unpredictably and since they are more widely expressed than their established roles would suggest (Table 1), it is likely that they have other poorly characterized functions.^13^ These receptors appear to bind several endogenous melanocortin agonists, the products of POMC gene transcription, as well as antagonists (such as agouti, agouti signalling protein [ASIP] and agouti related protein) but with inconsistent relative affinities and effects.^13^ It is possible that post‐translational modifications determine receptor localization within the nanodomains.^13^, ^14^ Within each nanodomain there will be a variety of proteins, including ion channels, modifying proteins and other GPCRs, that can interact with the MCRs to alter the availability of receptor at the cell surface as well as the intracellular signalling resulting from receptor activation.^13^ Different combinations of interacting proteins and MCRs may therefore give rise to the complex and inconsistent functional profiles reported for the MCRs.^13^

**TABLE 1:** Melanocortin receptors; tissue distribution, known agonists and antagonists, and their biological effects^1^, ^2^, ^3^, ^4^, ^5^, ^6^, ^8^, ^9^, ^10^

Receptor	Tissue distribution	Species	Agonist	Biological effects	Antagonist	Biological effects
MC1R	Melanocytes, adipocytes, keratinocytes, fibroblasts	Human	α‐MSH, ACTH, β‐MSH, γ‐MSH	Pigmentation, anti‐inflammatory role	Agouti	Suppresses melanin production
	Grey matter	Rat	NDP‐MSH	Anti‐inflammatory role		
			BMS‐470539			
	Monocytes, macrophages (including alveolar), lymphocytes, neutrophils	Human, murine	AP‐1189			
			
MC2R	Adrenal cortex, adipocytes; absent in liver, lung, thyroid and kidney	Rhesus macaque	ACTH	Stimulate steroidogenesis	GPS1574	Blocks steroidogenesis
	Chondrocyte and osteoblast	Human				
MC3R	Brain, placenta, duodenum, pancreas, stomach; absent in adrenal, kidney, liver	Rat	γ‐MSH ≥ ACTH = β‐MSH = α‐MSH	Energy balance, cardiovascular function	SHU‐9119	Inhibits anti‐inflammatory effects of γMSH
	Present in lung	Murine	MTII	Anti‐inflammatory		
			D‐Trp8‐γMSH			
	Macrophages and monocytes	Murine	AP‐214		AVM‐127	Inhibits α‐MSH‐induced penile erection
			AP‐1189 (biased agonist)			
MC4R	Brain, spinal cord, muscle; absent in lung, liver, kidney, adrenal	Rat/Canine	α‐MSH = ACTH > β‐MSH > γ‐MSH	Energy balance, erectile function, cardiovascular effects	AgRP	Inhibitory cardiovascular effects, increases food intake
	Hypothalamus and thalamus	Rat	THIQ	Anti‐inflammatory, inhibits food intake	ML00253764	
			Ro27‐3225			
			PT‐141	Induces erection		
MC5R	Brain, sebaceous glands, lung, skeletal muscle, adrenal, spleen, testis, ovary, muscle, adipocytes, mast cells; absent in adrenal	Murine/Human	α‐MSH, ACTH, β‐MSH,	Anti‐inflammatory		
	B‐lymphocytes	Mouse				
	T‐lymphocytes	Mouse	PG‐901	Inhibits glucose uptake		

Abbreviation: α‐MSH, α‐melanocyte‐stimulating hormone.

There are a number of synthetic MCR agonists and antagonists, but these will not be discussed in much further detail.^17^, ^18^

### 2.3 POMC, obesity, pigmentation and skin disease

Mouse knockouts (ko) for *POMC*, *MC4R* and *MC3R* all show an obese phenotype, as do humans expressing mutations of *POMC* and *MC4R*.^3^, ^19^, ^20^, ^21^, ^22^ Human subjects with specific mutations in *βMSH* have been found to be obese too, as have mice with engineered β‐endorphin deficiency and β‐MSH may indeed be mediating the satiety signal triggered by peripheral mediators crossing the blood‐brain barrier.^3^, ^12^, ^19^, ^23^ The CNS POMC system has other functions, including coordinating the central stress response, regulation of sexual behaviour, growth, lactation, the reproductive cycle and possibly central cardiovascular control.^3^, ^5^, ^12^, ^19^, ^22^ Transcriptional regulation of the POMC gene within the arcuate nucleus may generate these diverse functions.^24^ For example, the transcriptional regulator *PRDM12* is critical for *POMC* gene expression in the mouse arcuate hypothalamus in controlling food intake, adiposity and body weight.^24^ *PRDM12*, which is identified as a highly expressed gene in this arcuate neuronal population, plays an important role in the identity and function of POMC neurones.^24^ Its absence in adult mice greatly impairs *POMC* expression and leads to increased food intake and obesity.^24^ Single‐cell sequencing of arcuate POMC neurones has shown that they are neuroanatomical a highly heterogenous population, supporting the findings of possibly multiple differential transcriptional inputs.^25^ The main signal to POMC in the arcuate nucleus is from leptin, released from fat stores, which then crosses the blood‐brain barrier to inhibit feeding.^26^ There are other non‐arcuate pathways mediating appetite, for example, the mesencephalic trigeminal nucleus controls food intake and body weight via hindbrain POMC projections.^27^

**FIGURE 4:**
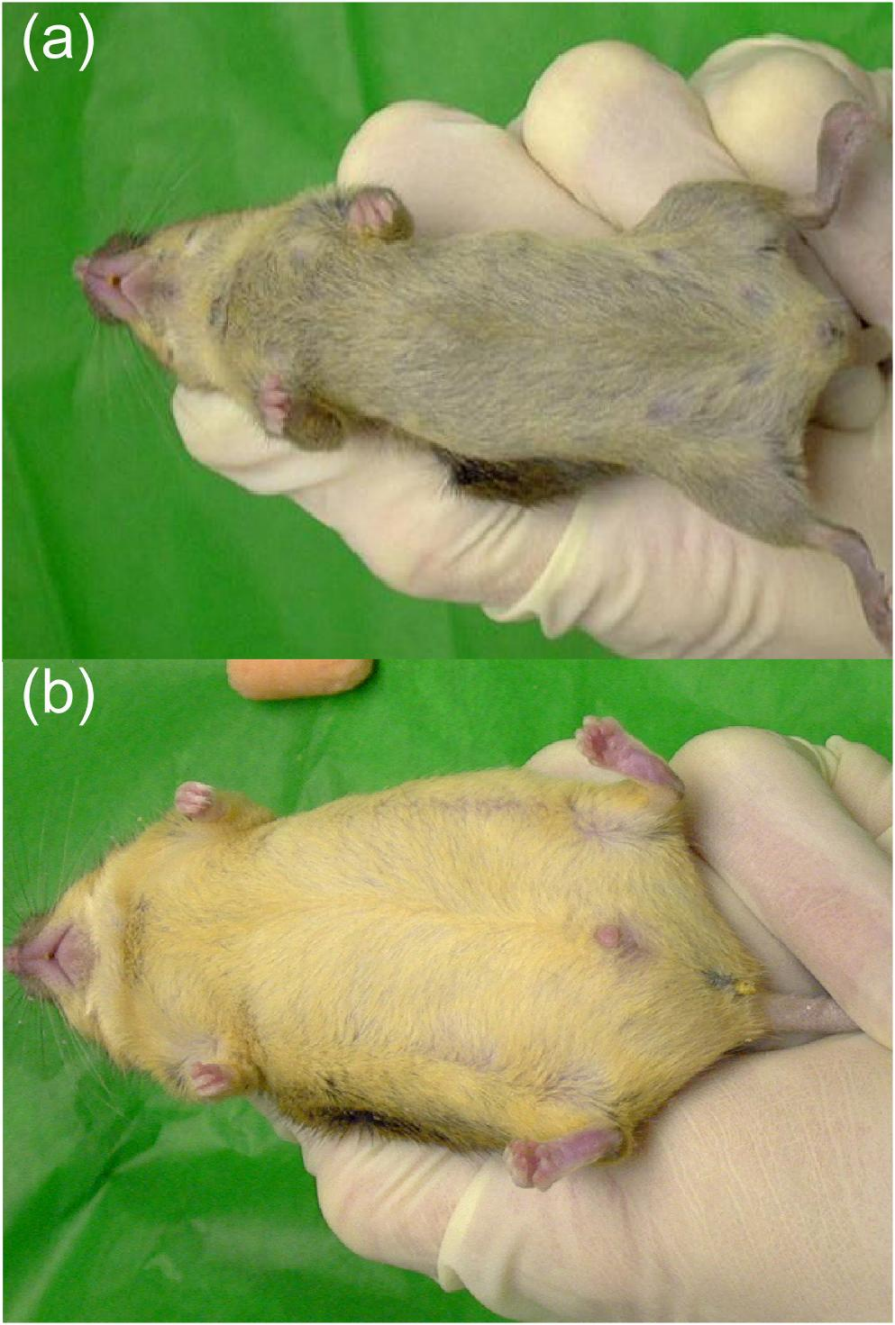
(a) Wild‐type mouse (+/+). (b) POMC homozygous mutant mice (−/−) become obese and develop a lighter hair colour on the ventral surface than sex and age matched wild‐type littermates (a). POMC, proopiomelanocortin.

Mutations in the MC1R gene lead to fair skin and red hair in humans,^4^, ^10^, ^28^ which is also seen with the inactivating human *POMC* and *PC1/PC3* genes.^29^ (Figure 4).^22^ mutations.^19^, ^20^, ^21^ Polymorphisms associated with loss of function of MC1R are also correlated with an increased incidence of the three commonest forms of skin cancer.^4^, ^10^, ^28^ Other mutations can occur in the *POMC* system or parallel interacting pathways, such as in prohormone convertase 1 and 3 (which also leads to obesity) and ASIP, a human homologue of murine agouti protein.^3^, ^10^, ^19^, ^20^, ^21^, ^22^ However, they do not necessarily affect skin colour, function or metabolism in humans, and further studies are needed.^3^, ^19^, ^20^, ^21^, ^22^ Two sisters were described recently with a novel POMC gene variant, leading to an ACTH defect, functionally via an altered prohormone convertase 2 cleavage site (Figure 2).^14^ The patients had obesity, hyperphagia and hypoadrenalism, with markedly elevated levels of ACTH, but unaffected pigmentation.^14^ Functional studies of this variant imply that their ACTH has decreased effectiveness at stimulating the melanocortin MC2R, leading to low levels of circulating cortisol.^14^ The hyperphagia and obesity provide evidence that adequate cleavage of ACTH to α‐MSH and desacetyl‐α‐MSH is also required in humans for feeding control, but may not be necessary for melanogenesis.^14^ A novel MC4R agonist, setmelanotide, has shown promising results with regard to weight loss in patients with POMC deficiency.^15^ A loss of function mutation in the MC4R is associated with obesity, tall stature and is more recently recognized to be associated with delayed gastric emptying.^16^

Ultraviolet (UV) absorption by the skin not only triggers mechanisms that defend skin integrity and regulate global homoeostasis but also induces skin pathology (e.g., cancer, ageing, autoimmune responses).^10^ Ultraviolet (UV) radiation can upregulate local neuroendocrine cutaneous systems, especially via UVB.^10^ The locally induced cytokines, CRF, urocortins, POMC peptides, enkephalins and others may be released into the circulation to exert systemic effects, including activation of the central HPA axis, opioidogenic effects and immunosuppression, independent of vitamin D synthesis.^1^, ^2^, ^10^ These pathway may contribute to the immunosuppressive effects of UV radiation.^10^ Although absolute Mendelian *POMC* deficiency is exceedingly rare, *POMC* variants in genome‐wide association studies are commonly associated with regular obesity.^19^, ^20^, ^21^ This supports the evidence that heterozygous variation in the *POMC* gene could be contributing to obesity in the human population relatively commonly.^19^, ^20^, ^21^, ^29^ However, the only psychological and dermatological consequences of impaired POMC function are perhaps impaired sexual function and an increased risk of skin cancer.^3^, ^4^, ^19^, ^20^, ^21^, ^22^

This is quite different for Cushing's syndrome, due to excess ACTH or exogenous glucocorticoids, characterized by central obesity, psychiatric disturbance, hypertension, diabetes mellitus, skin thinning, purpura and hyperpigmentation.^30^ Addison's disease (acquired deficiency of the HPA axis, specifically adrenal CSs), when endogenous, also typically presents with generalized hyperpigmentation.^31^ There are isolated cases of it presenting with psychosis.^31^

Simple obesity is extremely common^21^ and has many associations with skin diseases,^32^, ^33^ many of which have an immunological pathogenesis (Table 2).^34^ Obesity in itself is frequently associated with psychiatric disease and disruption of the POMC system may play a part in the psychiatric components, whether obesity is present or not.^35^, ^36^, ^37^ It seems reasonable to assume that either acute or chronic stress might be associated with flares of skin disease,^1^, ^2^, ^3^, ^10^ either through direct neuroendocrine effects,^1^, ^2^, ^3^, ^10^ epigenetic effects^38^ or through effects on the cutaneous immune system and microbiome.^4^, ^10^, ^28^, ^34^, ^38^

**TABLE 2:** Skin complications of obesity where immune dysfunction may be relevant

Alterations in the cutaneous microbiome Poor wound healing Cutaneous infection Lymphovascular disorders and ulceration Psoriasis Intertrigo Atopic eczema Hidradenitis suppurativa Pilonidal sinus Irritant contact dermatitis Scleroedema Livedo reticularis

### 2.4 Psychodermatology—Stress and the skin in general

The skin and the brain are intrinsically linked from early human development and it is perhaps not surprising that a disruption in the function of one will lead to disturbance in the function of the other.^39^, ^40^, ^41^ Existing with a dermatological condition presents constant functional, social, and psychological barriers for males and females alike.^40^ This has the real likelihood of impacting on quality of life.^40^ Thus, psychosocial interventions must be made more widely available to promote healthy coping strategies for living with skin conditions.^41^ A lot of the source of mental stress may relate to chronic stigmatization of the affected individual and this does not appear to be disease‐specific.^41^ This suggests that these effects could relate to activation of the HPA axis, with acute stress activating immune response (‘fight or flight’) and chronic stress altering the immune system's response to further stressors.^1^, ^2^, ^35^, ^36^, ^37^, ^42^ Chronic stress causes alterations in mood and feeding behaviour, as typical of endogenous depression.^43^ However, the neuroanatomical circuits associated with chronic stress and depression are not fully understood.^43^ A mouse model of chronic restraint stress produced hyperactivity of POMC neurones in the arcuate nucleus (cell‐bodies) of the hypothalamus (POMC^ARH^ neurones).^3^, ^19^, ^20^, ^21^, ^22^, ^43^ This was associated with decreased neural transmission in dopamine neurones in the ventral tegmental area (DA^VTA^ neurones).^43^ This study also showed that POMC^ARH^ neurones project to the VTA and provide an inhibitory tone to DA^VTA^ neurones.^43^ These results suggest POMC circuits, originating in arcuate nucleus of the hypothalamus, regulate feeding and mood in response to chronic stress, via inhibition of dopaminergic transmission.^43^ This could be a model linking POMC with neurological and psychiatric disease, some of which may be linked to skin disease.^43^, ^44^

Diseases associated with increased cellular immunity, however, seem to show exacerbation with acute stress (for example, psoriasis).^42^ From now on, this review will reflect predominantly on the effects of stress on 5 common dermatoses, namely psoriasis, atopic eczema (AE) (atopic dermatitis), acne, alopecia, vitiligo and melasma, as well as skin pigmentation and interaction with stress, pregnancy and the menstrual cycle on the skin.

### 2.5 Psoriasis, the HPA axis and psychodermatology

In in vitro studies from lesional skin, compared with healthy skin, the expression of genes of the cutaneous CRF‐POMC system and enzymes of melanogenesis^4^, ^10^, ^28^ are modified in psoriasis.^45^ The up‐regulation of POMC, CRFR1 and MCHR1 in the lesional and non‐lesional skin of patients with psoriasis is in line with a potential functional role for the local CRF‐POMC system^4^, ^10^, ^28^ in the pathogenesis of psoriasis.^45^ Up‐regulation of MCR1 in non‐lesional and lesional skin and decreased expression of ASIP (agouti) and enzymes of melanogenesis in the lesional and non‐lesional skin^4^, ^10^, ^28^ probably indicates the existence of a compensatory system^4^, ^10^, ^28^, ^42^ to inhibit the production of proinflammatory factors in the skin of patients with psoriasis.^42^, ^45^ In a follow on study looking at a range of single‐nucleotide polymorphisms (SNPs) in skin biopsies from patients with psoriasis, SNPs in the cutaneous CRF‐POMC system genes were positively correlated with plaque psoriasis.^46^ Epidermal keratinocytes in culture also express functional parallels of the cutaneous HPA system.^47^ Prolonged incubation or addition of calcium was associated with significant changes in the immunocytochemical expression of the cutaneous HPA system.^47^ Expression of CRF was more pronounced in less differentiated keratinocytes.^47^ POMC expression was enhanced in more differentiated keratinocytes.^47^ In all three of these in vitro experiments,^45^, ^46^, ^47^ it is not possible to say whether the effects in humans would relate to the actions of the peripheral cutaneous CRF‐POMC system,^4^, ^10^, ^28^ or the effects of circulating ACTH and the actions of the HPA system more generally.^1^, ^2^, ^6^, ^7^ To draw further conclusions would require in vivo^48^ and clinical studies.^49^

The physical and psychological symptoms and signs of psoriasis affect all parts of a patient's life.^50^ The psychological impact of the condition is frequently under recognized by clinicians. For example, the prevalence of depression and anxiety in psoriasis is significantly higher than that observed in the general population.^50^ Also, these affective disorders may even be linked to an increase in the severity of the physical illness.^50^ Increased HPA axis activity is seen during chronic stress,^1^, ^2^, ^3^, ^4^, ^5^, ^6^, ^7^, ^10^, ^11^, ^12^, ^35^, ^36^, ^37^, ^42^, ^51^ which also plays a key role in the pathophysiology of depression.^51^ Overactivity of the HPA axis occurs in major depressive disorder, leading to cognitive dysfunction and reduced mood.^51^ There is also a correlation between HPA axis activation and alterations in the gut microbiome, which could have a significant impact on the development of depression.^51^ Could alterations in the HPA axis explain the tendency of psoriasis patients to become depressed?^51^

A recent systematic review has shown a correlation between excess weight gain in psoriasis and worsening psychosocial outcomes.^52^ This is perhaps not surprising and fits with the previous discussions about obesity, depression and HPA function.^3^, ^12^, ^19^, ^20^, ^21^, ^22^, ^23^, ^24^, ^25^, ^26^, ^27^, ^29^, ^30^, ^32^, ^33^, ^34^, ^43^, ^50^, ^51^

There are wide variations in clinical outcomes in psoriasis.^53^ In a cluster analysis of health literacy, a significant subgroup had lower psoriasis knowledge, quality of life, ability to self‐manage and feelings of worth.^53^ There was a much smaller cohort where the opposite was true, suggesting dichotomy in populations.^53^ There is evidence of genetic variability in an individual's abilities to manage stress and this involves genes in the HPA axis.^54^

There is some evidence that psoriasis and AE may coexist in individuals. This could make interpreting studies involving both these conditions more complicated.^55^

Phototherapy is used in the treatment of psoriasis, including psoralen plus UVA (PUVA), broadband UVB (BB‐UVB) and narrowband UVB (NB‐UVB).^56^ NB‐UVB is considered safer and more effective than PUVA and BB‐UVB.^56^

### 2.6 AE, the HPA axis and psychodermatology

α‐MSH can elicit itch (or pruritus) responses too in mouse models.^57^ The biology of itch is complex, and it can occur both with a dermatosis and without.^58^ Stress and other psychological factors may play a part.^58^ In the skin of AE patients and mice with a model of AE, α‐MSH and PC2 are distributed mainly in the keratinocytes.^57^ In the skin of mice with a model of AE, MC1‐Rs and MC5‐Rs were expressed at the mRNA level, within the dermis.^57^ mRNAs encoding MC1‐R, MC‐3R, and MC5‐R were also located in the dorsal root ganglion (DRG) of mice with a model of AE.^57^ The naturally‐occurring MC1‐R antagonist ASIP^3^, ^10^, ^19^, ^20^, ^21^, ^22^, ^57^ inhibited spontaneous scratching in mice with a model of AE.^51^ In healthy mice, intradermal α‐MSH produced pruritus‐like responses, which were blocked by the thromboxane (TX) A2 receptor‐antagonist ONO‐3708.^57^ α‐MSH increased the production of TXA2 in mouse keratinocytes.^57^ This was subsequently inhibited by administration of the adenylyl cyclase inhibitor SQ‐22536 and the Ca2þ chelator EGTA.^57^ In mouse keratinocytes treated with siRNA for MC1‐R and/or MC5‐R, α‐MSH‐stimulated TXA2 production was reduced.^57^ α‐MSH increased intracellular Ca2þ ion concentration in DRG neurones and keratinocytes.^57^ These results suggest that α‐MSH is involved in generating pruritus during AE and may elicit itching through the direct action of primary afferents and TXA2 production by keratinocytes.^57^ These data may also provide part of an explanation for the therapeutic benefit of phototherapy in generalized pruritus and pruritic dermatoses.^4^, ^10^, ^57^, ^58^

CSs are effective anti‐inflammatory agents.^59^ The secretion of CSs is mostly controlled by the central HPA axis.^1^, ^2^, ^3^, ^4^, ^5^, ^6^, ^7^, ^8^, ^9^, ^10^, ^19^, ^20^, ^21^, ^22^, ^23^, ^24^, ^25^, ^26^, ^27^, ^42^, ^59^ However, they are also secreted locally by peripheral tissues, including the skin.^4^, ^10^, ^11^, ^13^, ^14^, ^15^, ^16^, ^17^, ^18^, ^19^, ^20^, ^21^, ^22^, ^28^, ^42^, ^59^ Several tissues express 11β‐hydroxysteroid dehydrogenase 1 (11βHSD1).^59^ The inactive CS cortisone is converted by 11β‐HSD1 to the active CS cortisol, which is responsible for the delayed wound healing and psychological problems seen as some of the established side‐effects of CS excess.^59^ However, the exact role of 11β‐HSD1 in inflammation is unclear.^58^ This study examined whether 11β‐HSD1 affected the development of AE in vitro and in vivo.^59^ The expression of 11β‐HSD1 in the epidermis of AE lesions was higher than in that of the epidermis of healthy controls.^59^ Knockdown of 11β‐HSD1 in human epidermal keratinocytes increased the production of thymic stromal lymphopoietin.^59^ Thymic peptides, such as lymphopoetin, are known to play an important role in feedback in the HPA system, leading to activation or inactivation of the HPA axis and consequent stress‐related behaviours.^6^, ^7^ In an oxazolone‐induced murine model of AE, localized inhibition of 11β‐HSD1 aggravated the development of AE and increased serum cytokine levels associated with AE, again leading to activation of the HPA axis and increased stress‐related behaviours.^1^, ^2^, ^42^, ^43^, ^59^ 11β‐HSD1 knockout mice developed significantly worse AE following induction with oxazolone, compared to wild‐type.^59^ They proposed that 11β‐HSD1 was a major factor affecting AE pathophysiology via suppression of atopic inflammation due to the modulation of active CS in the skin, with secondary interference of the central HPA axis.^1^, ^2^, ^6^, ^7^, ^42^, ^43^, ^59^

In a clinical cross‐sectional study in children aged 6–12 years, a diagnosis of attention deficit/hyperactivity disorder (ADHD), with or without associated AE, correlated with a reduced HPA axis response to an acute stressor.^60^ In this study, there was no evidence of significantly altered HPA axis activity in AE, nor of a significant interaction between AE and ADHD.^60^ This was discussed in view of the limited variation in AE symptom severity in this sample.^60^ However, in children with AE, HPA axis function was linked to (clinical and subclinical) ADHD symptomatology with specific impact on inattention and impulsivity, while no such associations were observed in children without AE.^54^ This observation underlines the possible major role of HPA axis pathophysiology in the coexistence of both conditions.^60^ Future studies are needed to add to the reported results and further explore the separate and joint pathophysiology of HPA axis function in both ADHD and AE.^60^

### 2.7 Acne, the HPA axis and psychodermatology

POMC, its by‐products ACTH, α‐MSH, β‐MSH and β‐endorphin are located in sebocytes, as well as the PC enzymes which are responsible for the cleavage of POMC.^61^ MC1‐R, MC2‐R and MC5‐R are also expressed in sebocytes, presumably with a functional role in the control of sebaceous differentiation and lipid synthesis.^61^ The melanocortins and ACTH induce differentiation and lipogenesis in sebocytes and reduce IL‐1βinduced IL‐8 secretion.^61^ Also, μ‐opioid receptors are located in sebocytes, soβ‐endorphin may also stimulate lipogenesis.^61^ MC5‐R was only found in differentiated sebocytes, suggesting perhaps a more specific role.^61^ The expression of the HPA axis in sebaceous glands suggests the presence of a local endocrine ‘stress axis’, which might be involved in cutaneous/sebaceous stress responses.^61^ This hypothesis is supported by findings that CRF expression is increased in sebaceous glands of acne‐affected skin and also in aged skin.^61^ Like CRF, MC1‐R also has increased expression in acne‐affected skin too.^61^ There have been a number of more clinical and psychosocial studies supporting the role of stress and mental health in the pathogenesis of acne too.^62^, ^63^

Against this argument of MC5‐R involvement in acne is a more recent study.^64^ Acne vulgaris is a multifactorial disease.^61^, ^64^ One of the main aspects that acts to influence acne pathogenesis is elevated sebum secretion.^61^, ^64^ Sebocyte differentiation followed by sebogenesis is essential for sebum secretion.^61^, ^64^ Sebocyte differentiation and growth, and sebum synthesis are controlled by complex pathways.^61^, ^64^ Studies have shown that perilipin 2 and MC5‐R play a role in sebogenesis.^61^, ^64^ Perilipin 2 is a protein which belongs to a family of cytoplasmic lipid droplet‐binding proteins.^64^ This protein surrounds the lipid droplet with phospholipids and is involved in aiding the storage of neutral lipids within the lipid droplets.^64^ The aim of this study was to see whether levels of perilipin 2 and MC5‐Rs correlate with the development of acne vulgaris.^64^ The study included 65 patients diagnosed with acne and 43 healthy control subjects.^64^ Perilipin 2 and MC5‐R levels were measured from blood samples, via the enzyme‐linked immunosorbent assay technique.^64^ No significant differences were observed between the acne group and the control group in serum perilipin 2 (*p* = 0.594) and MC5‐R (*p* = 0.213) levels.^64^ In the moderate acne group, perilipin 2 and MC5R levels were significantly higher than in the mild acne group (*p* = 0.0014, *p* = 0.003).^64^ The levels in the severe acne group were not higher compared to the moderate and mild acne groups.^64^ This study failed to detect any association between acne pathogenesis and perilipin 2 and MC5R serum levels, except when comparing moderately severely affected with mildly affected with acne.^64^ These proteins may have a mild influence on acne severity at best.^64^ It is noteworthy that the effects of psychosocial stress were not studied within the acne groups in this study.^64^ Also, the proteins were being measured indirectly from blood samples, rather than from a skin sample and the sample sizes involved in the study were rather small.^64^

### 2.8 Alopecia, the HPA axis and psychodermatology

CRF is recognized as an inhibitor of hair shaft production and it also is important in the premature stimulation of the catagen phase.^65^ CRF also has an influence on the reduction in the increase of keratinocytes in the hair matrix and the induction of their apoptosis.^65^ CRF may influence hair growth both directly and indirectly, predominantly through the upregulation of POMC gene expression and POMC processing in human hair‐follicles (HFs).^65^ In vivo studies on mammalian HFs suggest that ACTH induces the anagen phase by influencing steroid metabolism in the skin.^65^ The role of ACTH in human hair growth remains unclear.^65^ Hair cortisol concentration may be a promising diagnostic instrument in clinical practice and could have an impact on improving hair growth.^65^ The presence of cortisol in high levels is associated with a reduction in the synthesis and premature degradation of hyaluronans and proteoglycans, two important modulators of HF function.^65^ However, low cortisol levels can actually have positive effects on hair growth by slowing down the degradation of these two skin components.^65^

This ambiguous data about the effects of cortisol on hair growth in vitro, may translate into a dichotomy of opinion as to whether or not to use topical steroids in the clinical setting of alopecia areata.^66^ What is clear is that alopecia areata strongly correlates with psychological stress, perhaps leading to altered immune functioning.^65^, ^66^, ^67^

The exact purpose of POMC‐derived peptides in immunomodulation is still a large field requiring much further study.^1^, ^2^, ^4^, ^6^, ^7^, ^10^, ^11^, ^13^, ^14^, ^15^, ^16^, ^17^, ^18^, ^28^, ^67^, ^68^ The HF has an unusual immune system.^67^, ^68^ The proximal epithelium of an anagen HF is an area of ‘immune privilege’, characterized primarily by a very low level of expression of major histocompatibility (MHC) class antigens.^67^, ^68^ Inherent immune privilege deficiencies might confer increased susceptibility to alopecia areata for some individuals.^67^ In previous studies, α‐MSH was promoted as a promising therapeutic target for restoration of immune privilege.^68^ Follicular cells also produce ACTH and β‐endorphin.^68^ ACTH, β‐endorphin and α‐MSH, may all help in the regulation of the unique immune system of the HF.^67^, ^68^ Human occipital scalp skin specimens were obtained from hair transplantation surgery, cultures were isolated as described previously.^68^ This in vitro study, with human tissue, indicates that the α‐MSH, ACTH and β‐endorphin are promising contenders for immune privilege maintenance and restoration, through suppression of ectopic expression of MHC class I.^68^

Several reports have shown that CRF inhibits hair growth and induces hair loss.^69^ However, the underlying mechanisms are still ambiguous.^69^ This study looked at the effect of CRF on human dermal papilla cells (DPCs), as well as HFs, and to see whether the HPA axis was present in cultured human DPCs.^69^ CRF inhibited hair shaft elongation and induced early catagen transition in human HFs. HF cells, both human DPCs and human outer root sheath cells, expressed CRF and its receptors and responded to CRF.^69^ CRF inhibited the increase of human DPCs through cell cycle arrest at G2/M phase and stimulated the increase in reactive oxygen species.^69^ Anagen‐related cytokine levels were reduced in CRF‐treated human DPCs.^69^ CRF increased the activity of POMC, ACTH, and cortisol in human DPCs.^69^ CRF receptor antagonists blocked these actions of increased HPA activity on the DPCs.^69^ The results of this study suggest that stress can cause hair loss by acting through hormones of the HPA axis.^69^ The results may also suggest that a fully functional HPA axis exists in human DPCs and that CRF directly affects human DPCs, as well as human HFs, under conditions of stress.^69^

Androgenetic alopecia (AGA) is thought to be aggravated by stress too.^70^ CRF was used to induce a stress response in human ex vivo male AGA HFs.^70^ Caffeine reverses testosterone‐mediated hair growth inhibition in the same model of hair organ culture.^70^ The objective of the study was to see whether caffeine would block CRF‐mediated stress in this HF‐model system^70^ HFs from scalp biopsies of men affected by AGA were incubated with CRF plus or minus caffeine.^70^ When compared with controls, CRF stimulated the expression of TGF‐β2 (*p* < 0.001), CRF receptors 1 and 2 (*p* < 0.01), ACTH) (*p* < 0.001), MC2‐R (*p* < 0.001), and other stress‐associated substances, such as substance P (*p* < 0.05) and p75 neurotrophin receptor (*p* < 0.01).^70^ CRF also inhibited matrix keratinocyte proliferation and expression of both IGF‐1 and the pro‐proliferative nerve growth factor receptor, NGF‐tyrosine kinase receptor A (tyrosine kinase receptor A).^70^ Caffeine significantly antagonized all the previously described effects of CRF, whether HPA related or not, in ex vivo human AGA HFs.^70^

In a more clinical study, plasma concentrations of free cortisol and ‘MSH’ were compared between 43 healthy subjects and their 37 controls.^71^ They found no significant difference between the alopecia areata group and the control group for both ‘MSH’ and cortisol levels.^71^ The cortisol levels were noticeably higher (but not significantly) in the alopecia areata group and it is tempting to think they would have had a different result if they had studied bigger populations.^71^ ‘MSH’ in this study is presumably α‐MSH, but this is not stated.^3^, ^4^, ^10^, ^11^, ^13^, ^14^, ^15^, ^16^, ^17^, ^18^, ^72^ Finally, it is very difficult to measure the levels of the individual melanocortins accurately (e.g. the MSHs), because they are structurally very similar (Figure 2).^3^, ^4^, ^72^ ACTH, β‐endorphin and POMC are sufficiently larger and structurally different to be measured more reliably.^3^, ^4^, ^72^

### 2.9 Vitiligo, the HPA axis and psychodermatology

Vitiligo is a common depigmenting skin disorder with an estimated prevalence of 0.5%–2% of the population worldwide.^73^, ^74^, ^75^ It was first described more than 1500 years BC.^73^, ^74^ Both pre–Hindu Vedic and ancient Egyptian texts give a clear record of depigmented macules.^73^, ^74^ The Vedic myth was that the anthropomorphic deification of the sun, Bhagavantam, developed vitiligo after being gazed upon by his illegitimate son.^73^, ^74^ The curse of vitiligo in certain populations from South Asia, of all religious backgrounds, is still a problem, so that some women with the disease might still be considered unmarriageable even now.^73^, ^74^ In certain Hindu texts, a person who is said to have committed a particular offence of insulting a religious teacher (guru droh) in a previous life, suffers vitiligo in the next life.^73^, ^74^ Vitiligo is clearly a disease with a long history of Psychodermatological issues.^73^, ^74^

A recent in vitro study, which included 40 vitiligo patients and 40 controls, looked at a possible role for the melanocortin system in the pathogenesis of vitiligo.^75^ Skin biopsies were taken from both lesional and non‐lesional skin of patients and from the non‐solar exposed skin of controls.^75^ Quantitative real‐time PCR was used to detect the expression of *POMC* and *MC1‐R*.^75^ The expression of both *POMC* and *MC1‐R* were significantly lower in the lesional skin, compared with both the non‐lesional skin of vitiligo cases, as well as compared with the controls, while they were significantly higher in non‐lesional vitiligo skin than in controls.^75^ In conclusion, changes to the POMC system could be the end result of the disease.^75^

Management of vitiligo includes the use of drugs, cutaneous transplant and phototherapy.^76^ NB‐UVB can activate melanocytes to proliferate and mobilise, ultimately migrating to the epidermis and resulting in repigmentation of vitiligo skin lesions.^76^, ^77^

### 2.10 Melasma, the HPA axis and psychodermatology

Melasma has not always been considered as psychocutaneous disorder, perhaps because it is commoner in South Asians and therefore has not been reported as much in the literature.^78^ A recent cross‐sectional study from India considered 50 consecutive patients with melasma between the ages of 18 and 65 years^78^ Those with vitiligo or any other physical skin condition were excluded.^78^ Those with a history of significant sun exposure, current pregnancy or existing oral contraceptive pill usage were excluded too.^78^ The final exclusion criterion was that clinically significant intellectual or cognitive impairment could not be included.^78^

These melasma cases were compared with 30 controls.^78^ Assessments of stress, anxiety and melasma, using standardized international proformas, were performed by a psychiatrist and dermatologist respectively.^78^

The study found high psychiatric comorbidity (76%) in patients with melasma, with most of the patients suffering from depression (42%).^78^ Significantly higher anxiety and depression scores were found in melasma patients than the comparable control group.^78^ There were significant impairments in functioning, which were correlated statistically with the severity of psychiatric disease, but not with severity of melasma.^78^ Patients presenting with melasma should therefore be assessed for psychological and psychiatric disease.^78^ Unlike the previous skin disorders mentioned, the evidence for POMC and the HPA axis having a role in the pathogenesis of melasma is more limited.^4^, ^10^, ^78^, ^79^, ^80^

### 2.11 Skin pigmentation in general, the HPA axis and psychodermatology

POMC, via ACTH and α‐MSH, are amongst the principle neuroendocrine factors affecting skin pigmentation, through actions on the MC1‐R in melanocytes.^4^, ^10^, ^11^, ^13^, ^14^, ^15^, ^16^, ^17^, ^18^, ^20^, ^21^, ^22^, ^28^, ^29^, ^31^, ^80^ For millennia, Europeans have discriminated against those with darker skins, often Africans.^81^ This was sometimes institutionalized in the form of very flawed academic study of human pigmentation.^81^ Unfortunately, humans do not learn easily from history and make very similar mistakes now. For example, sunbed use is an addictive behaviour and the mechanisms for dependence may be through central release of the POMC peptide β‐endorphin.^82^ Rather worryingly, a study from Denmark found that in 2008, there was significant use of sunbeds in under 18's and this included children as young as 8.^83^

A survey of 4703 people in Italy investigated the risk factors associated with sunbed use.^84^ Sunbed use was significantly higher among women, the under 35's and more educated people, defined as beyond high school education.^84^ Subjects at particularly high risk of melanoma and other skin cancers used sunbeds significantly more; for example, people with freckles, red hair or blue/green/grey eyes.^84^ Polymorphisms associated with loss of function of MC1R are associated with an increased risk of photoageing, freckles, red‐hair and all types of common skin cancers, independent of these behavioural variables.^4^, ^10^, ^11^, ^13^, ^14^, ^15^, ^16^, ^17^, ^18^, ^20^, ^21^, ^22^, ^28^, ^29^, ^31^, ^80^, ^81^, ^85^, ^86^

The reinforcing effects of frequent tanning may be explained by the role of endorphins in the physiologic response to the UV exposure of sunbeds.^87^, ^88^ In a randomized controlled trial, frequent sunbed users demonstrated a preference for UV‐emitting sunbeds over non‐UV‐emitting sunbeds in a blinded trial.^87^ The administration of naltrexone as an opioid blockade reduced this UV preference, and was found to induce withdrawal symptoms.^87^ This suggests a mechanism in frequent tanning which is consistent with opioid‐dependency.^87^ Cutaneous b‐endorphin levels are also higher post‐UV exposure in frequent tanners, which may explain the role of endorphins in the preference of frequent tanners for UV‐emitting sunbeds.^88^

At the other end of the spectrum is the practice of skin‐lightening. This a common practice in some parts of the world, as illustrated by this study from Nigeria.^89^ Adverse effects from skin‐lightening products (SLP) are a significant public health concern.^89^ The aim of the study was to assess the prevalence, determinants and perception of use of SLP among female undergraduate medical students in Nigeria.^89^ This was a cross‐sectional descriptive study among female medical students as an on‐line survey, using a semi‐structured self‐administered questionnaire containing participants' biodata, history, pattern and perception of use of SLP.^89^ A total of 110 respondents completed the survey with over half (62; 56.4%) of them ranging between 20 and 24 years of age.^89^ The prevalence of SLP use was 45/110 (40.9%) with facial cleansers being the commonest product used (23/45 [51.1%]).^89^ Over 80% of the participants knew SLP could cause adverse effects with skin irritation being the most commonly mentioned (71/110 [64.5%]). Although most (80%) respondents did not think that light skin was superior to dark skin, removal of discolouration/dark spots (40%) and other cosmetic motives (37.8%) were the commonest reasons for use.^89^ Determinants of use of SLP were light skin colour (OR 3.8, 1.572–9.318), history of use among relatives (OR 3.3, 1.384–7.793) and awareness of adverse effects (OR 3.3, 1.129–9.740).^86^ What is interesting about this study^86^ and the sunbed study from Italy^83^ is that it may be that educated people, perhaps particularly, are bothered about skin colour issues.^83^, ^89^ This would need further study.

Obsessive tanning or skin‐lightening behaviour could be said to represent dysmorphophobia, a non‐acceptance with one's appearance, which often correlates with a history of depression and anxiety.^90^ Depression may be triggered by chronic stress, via activation of CNS POMC pathways.^44^ Another factor in the POMC pathway, MC1R is mutated in individuals with red hair and freckles, the most likely to use sunbeds.^4^, ^10^, ^11^, ^13^, ^14^, ^15^, ^16^, ^17^, ^18^, ^20^, ^21^, ^22^, ^28^, ^29^, ^31^, ^80^, ^81^, ^85^, ^86^ Therefore both peripheral and central alterations in the melanocortin‐POMC pathways may interact to contribute to pathological psychological reactions to skin pigmentation.^4^, ^10^, ^11^, ^13^, ^14^, ^15^, ^16^, ^17^, ^18^, ^20^, ^21^, ^22^, ^28^, ^29^, ^31^, ^43^, ^80^, ^81^, ^85^, ^86^

## 3 CONCLUSIONS

While the role of POMC has been studied in the pathophysiology of a number of dermatoses, there is no absolute evidence as yet that it plays a role in mediating the effects of stress in all skin diseases. Nevertheless, there is increasing indirect evidence of alterations in both the central and peripheral POMC systems linking both behaviour and certain cutaneous diseases, suggesting POMC could be a key molecule in Psychodermatology, connecting brain and skin.

## CONFLICT OF INTEREST

GWMM is the Editor‐in‐Chief of Skin Health and Disease.

## AUTHOR CONTRIBUTIONS

**George W. M. Millington**: Conceptualization (Equal); Data curation (Equal); Formal analysis (Equal); Investigation (Equal); Methodology (Equal); Project administration (Equal); Supervision (Equal); Writing – original draft (Equal); Writing – review & editing (Equal). **Hannah E. Palmer**: Conceptualization (Equal); Formal analysis (Equal); Funding acquisition (Equal); Project administration (Equal); Writing – review & editing (Equal).

## ETHICS STATEMENT

Not applicable.

## Data Availability

Data sharing is not applicable to this article as no new data were created or analyzed in this study.
